# Streamlining oversight in Medicare advantage: measuring coding intensity via contract rankings

**DOI:** 10.1093/haschl/qxag209

**Published:** 2026-09-11

**Authors:** Trenton Chang, Michelle Qiu, Zhiyi Hu, Dylan Zapzalka, Divya M Shanmugam, Daniel K Shenfeld, Ravi B Parikh, Maggie Makar, Ezekiel J Emanuel, Zach Brown, Amanda E Kowalski, Jenna Wiens

**Affiliations:** Division of Computer Science and Engineering, University of Michigan, Ann Arbor, MI 48109, United States; Division of Computer Science and Engineering, University of Michigan, Ann Arbor, MI 48109, United States; Division of Computer Science and Engineering, University of Michigan, Ann Arbor, MI 48109, United States; Division of Computer Science and Engineering, University of Michigan, Ann Arbor, MI 48109, United States; Department of Computer Science, Cornell Tech, New York, NY 10044, United States; Department of Medical Ethics and Health Policy, University of Pennsylvania, Philadelphia, PA 19104, United States; Winship Cancer Institute, Emory University, Atlanta, GA 30322, United States; Division of Computer Science and Engineering, University of Michigan, Ann Arbor, MI 48109, United States; Perelman School of Medicine, University of Pennsylvania, Philadelphia, PA 19104, United States; Department of Economics, University of Michigan, Ann Arbor, MI 48109, United States; National Bureau of Economic Research, Cambridge, MA 02138, United States; Department of Economics, University of Michigan, Ann Arbor, MI 48109, United States; National Bureau of Economic Research, Cambridge, MA 02138, United States; Division of Computer Science and Engineering, University of Michigan, Ann Arbor, MI 48109, United States

**Keywords:** Medicare Advantage, medicare, coding intensity, auditing, payment reform, payment policy

## Abstract

**Introduction:**

Risk-adjusted payments in Medicare Advantage (MA) incentivize insurers to maximize reported diagnoses, resulting in high coding intensity, a major source of Medicare overpayments. However, distinguishing justifiable coding intensity from misreporting is challenging: some approaches risk conflating coding intensity due to extraneous diagnoses with that due to variation in clinical practice or beneficiary health, while others assume specific overreporting mechanisms.

**Methods:**

We propose a data-driven method for ranking MA contracts by coding intensity using comparisons between geographically co-located contracts based on adjusted diagnosis rates among switchers from Traditional Medicare to MA. The approach is applied to a 20% sample of 2019 MA claims.

**Results:**

Health Maintenance Organization (HMO)-dominated contracts tend to rank higher than preferred provider option (PPO)-dominated contracts, echoing previous findings. Furthermore, highly ranked contracts administer procedures and prescriptions relevant to beneficiaries’ diagnoses less often than lower-ranked contracts, supporting the validity of the ranking. A simulation study suggests even an imperfect ranking can recover overpayments faster than a random audit, if mean diagnosis rates are low (10%) and vary widely across contracts.

**Conclusion:**

As MA looks to expand auditing strategies, MA enrollment continues to grow. The proposed approach can facilitate payer-specific audits even as mechanisms driving coding intensity evolve.

## Introduction

Over half of all Medicare beneficiaries, or 34.4 million individuals (2025), are enrolled in Medicare Advantage (MA).^1^ The Centers for Medicare and Medicaid Services (CMS) reimburses MA contracts a per-beneficiary, risk-adjustment payment (“capitation”) intended to cover costs of member care. Payments increase for patients of higher illness severity, as determined by Hierarchical Condition Categories (HCCs), or pre-defined groupings of diagnosis codes.^2^ While this system is intended to support care and mitigate selection against beneficiaries with greater medical needs, it creates a financial incentive for insurers to “game” the model by overreporting HCCs to maximize subsequent payments, leading to high “coding intensity.”^3^ Higher HCC scores due to high coding intensity are estimated to cost CMS about $40 billion annually in overpayments, straining the financial solvency of Medicare.^3,4^

CMS employs the Risk Adjustment Data Validation (RADV) audit as its primary oversight tool. RADV audits target contracts, a bundle of Medicare coverage options offered by Medicare Advantage organizations (MAOs), ie, insurers. These audits estimate improper payments on a small sample of enrollees in each contract and estimate payment error based on unsubstantiated diagnoses.^5^ However, the growing scale of the MA program presents a challenge to effective oversight. Given recent pushes to expand the scope of RADV, prioritizing contracts with high coding intensity for auditing could mitigate backlogs in the CMS oversight process.^6^

Prior work measuring coding intensity generally compares diagnosis patterns in Medicare Advantage to those expected based on proxies of patient health derived from Traditional Medicare (TM).^3,7-9^ Methods proposed by the Medicare Payment Advisory Committee (MedPAC) compute a score based on spending in TM accounting for age, sex, and dual-enrollment status, and others compute similar TM-derived baselines and assume any spending or excess risk score beyond the TM benchmark is inappropriate.^3,7^ However, estimating coding intensity in individual MA contracts is challenging, as differences in observed diagnosis rates may reflect a combination of coding practices, geographic variation in care patterns, and differences in beneficiaries’ underlying health.

Other approaches make assumptions about how coding intensity currently manifests. For example, examining whether diagnoses from chart reviews and in-home health risk assessments are associated with differences in the rate of specific treatments targeting reported diagnoses.^10^ Such approaches may become inaccurate if insurers adopt new strategies for maximizing coding intensity. Ultimately, without a resource-intensive audit, it is difficult to determine whether gaps in diagnosis rates correspond to reporting diligence or extraneous diagnoses.

This study introduces an approach for comparing coding intensity across MA contracts that makes use of within-market comparisons and prior-year TM diagnoses among TM-to-MA switchers to support contract-level audit prioritization. Higher HCC scores due to high coding intensity have long been recognized as a source of overpayments in MA, with CMS announcing its intent to audit all contracts.^6^ Given finite auditing resources, a high-quality contract ranking could improve the efficacy of overpayment recovery relative to alternative auditing strategies. This work adopts a switchers’ design, examining beneficiaries who switched from TM to MA, following prior work. Brown et al. leveraged TM-to-MA switchers to compare beneficiaries entering MA with those remaining in TM, using pre-switch health status to study how risk adjustment altered selection into Medicare Advantage by comparing expenditures between stayers and switchers.^11^ Jacobs and Layton used switchers to estimate coding intensity, comparing risk scores between stayers and switchers.^12^ In contrast, rather than comparing TM to MA, we use switchers to construct a common baseline for underlying beneficiary health to study contract-level variation in coding intensity within MA, producing a contract-level ranking of coding intensity.

The ranking is constructed using pairwise comparisons of diagnosis rates between geographically co-located contracts, adjusted for underlying health status, as captured by prior-year risk. These contract-to-contract comparisons are collapsed into a ranking of contracts by coding intensity. Several tests of the proposed ranking are performed to demonstrate internal consistency and partial external validity. Since the proposed ranking does not assume any mechanism of coding intensity, it remains applicable even under changes in insurer strategy in response to new CMS policies.^11^

## Methods and data

### Study cohort

The dataset consists of a 20% sample of MA beneficiaries in 2019. In contrast to current MedPAC practice, which examines growth in risk scores within concurrent TM and MA cohorts, this study examines switchers, or beneficiaries in 2018 TM who switched to MA in 2019. Using Medicare switchers controls for past MA diagnosis coding by ensuring that all beneficiaries are similarly exposed to baseline 2018 TM coding patterns. Within switchers, the study is restricted to continuing aged, non-dually eligible, non-ESRD community-based beneficiaries, since these beneficiaries are subject to the same risk-adjustment model, and thus similar diagnostic reporting incentives.

Further exclusions include beneficiaries who moved states, since MA offerings are geographically scoped, and those with any 2019 hospice claims, since hospice is not covered by MA. To ensure all switchers are similarly exposed to MA coding strategies, beneficiaries were only included if they (1) had a full year of TM in 2018, (2) were alive at the end of the study period (December 2019, as is the MedPAC practice), and (3) stayed with the same MA contract for all of 2019.^3^ Finally, beneficiaries in counties where only one contract is observed were excluded: since the proposed approach leverages local comparisons between contracts, such comparisons are ill-defined in one-contract counties. See Appendix Figure A1 for a cohort diagram.

The switchers’ cohort is also compared to MA stayers from 2018 to 2019 under identical exclusion criteria, since switchers may not be representative of the national MA population. Comparisons are performed using a Wilcoxon rank-sum test for continuous features and a chi-squared test for categorical features. See Appendix Table A2 for the comparison table.

### Data extraction

Claims eligible for risk adjustment based on the Version 22 HCC definition and 2019 eligible CPT codes are considered, yielding a subset of inpatient, outpatient, and carrier claims. We extract HCCs from diagnosis codes from 2018 TM claims and 2019 MA encounter files. For downstream validation, we use prescription information sourced from Part D event files. All data extraction and analysis were performed using open-source Python packages.

#### Statistical analysis

The proposed method outputs a ranking of MA contracts based on coding intensity using causally-motivated gaming detection, a technique to rank entities such as MA contracts that modify their behavior in response to incentives.^12,13^ The approach adjusts for differences in underlying patient health by stratifying beneficiaries on prior-year risk scores reported in TM and restricting comparisons to MA contracts in the same geographic “markets” to control for variation in regional practice patterns, where “markets” are defined as sets of counties offering identical MA contracts. Diagnosis rates are computed with respect to HCCs defined under the V22 CMS-HCC model, in use in 2019. To ensure stable estimates and avoid overinterpreting sparse diagnoses, we restrict the HCC set to those with a prevalence of at least 0.5% in the switchers’ cohort, yielding 45 eligible Version 22 HCCs (of 79 total; see Appendix Table A3).

### Estimating rankings

The proposed approach first estimates within-market rankings, then aggregates across markets. Markets are defined as sets of counties in which beneficiaries have the same choice of MA contracts. In each market, per-contract diagnosis rates are adjusted for differences in enrollee health by comparing contracts as if they enrolled a nationally representative population with respect to prior-year risk. Specifically, beneficiaries are stratified by prior-year CMS-HCC risk decile given reported ICD-10 codes, age, and sex in 2018 TM. Then, per-decile diagnosis rates are shrunk towards the market-level mean. Lastly, per-decile diagnosis rates are combined via a weighted average to reflect a common national risk distribution. This process yields a ranking of adjusted per-contract diagnosis rates.

To combine within-market rankings, we extract pairwise “matchups” between all contracts in a market and assign each matchup a weight based on the difference in HCC rates between contracts, market size (number of beneficiaries), and HCC model coefficient, which corresponds to the risk-adjusted payment associated with an HCC. These matchups are used to fit a Bradley–Terry model, a model of relative competitor strength suitable for producing global rankings in competitions with incomplete matchups.^14,15^ Intuitively, contracts that code at higher rates than their geographically co-located competitors should be ranked higher than those that code below their geographic peers. Furthermore, since the Bradley-Terry model assigns each contract nationally a single scalar parameter corresponding to its level of coding intensity relative to peers, rankings output by this procedure are directly comparable.

To account for variability, adjustment, ranking, and aggregation are re-run across 1000 within-market, within-contract bootstrap samples. The median and interquartile range (IQR) of the final rank of each contract is reported over all contracts in the cohort, anonymized to avoid any appearance of alleging malfeasance. To summarize patterns across the large number of contracts, the share of HMO (Health Maintenance Organization) and PPO-dominated (Preferred Provider Option) contracts within each decile of our ranking is also reported, given prior evidence of differences in coding practices across such plans.^9,16^ A detailed description of the ranking pipeline is in the Appendix. As a sensitivity analysis of the coding, we also repeated the analysis using CMS-HCC V28 HCCs in place of V22 HCCs.

#### Evaluating rankings

Validating rankings against ground truth is challenging as audits are sparse. This work conducts internal and external validity checks to verify that the rankings align with properties expected of a ranking consistent with detecting high coding intensity. As a sensitivity analysis of adjustment, the Kendall tau correlation between the proposed ranking and naive rankings is reported, based on unadjusted and adjusted average per-contract risk scores based on 2019 MA.^14^ Uncertainty in the ranks is compared to that of a random ranking to ensure the proposed approach is meaningfully differentiating contracts.

In addition, as a proxy for external validity, differences in the rate of relevant treatments (prescriptions or procedures) in the same year conditional on specific HCCs are analyzed, based on the assumption that legitimate diagnoses are associated with higher rates of relevant treatments.^10,15^ We analyze 5 conditions with sufficiently high prevalence and specific treatments: diabetes with complications, psychiatric conditions, arrhythmia/vascular COPD, and Parkinson's disease. HCC-to-treatment mappings are reported in Appendix Table A4. To address missingness in prescription drug data, this analysis is performed for beneficiaries with 12 months of Part D coverage.

#### Simulation studies

To assess the utility of rankings as a function of accuracy, a simulation study was performed. Specifically, we simulated how quickly contracts that code intensely would be identified using the proposed approach. Noise is added to the ranking to simulate rankings with varying levels of accuracy as measured using the c-index; see the Appendix for details.^17^

### Limitations

The choice to study a switchers cohort involves an important design tradeoff. While analyzing switchers enables straightforward adjustment for prior-year diagnoses unaffected by coding intensity incentives, switchers represent a minority of the MA population and may not be representative of a national cohort. While this decision may limit generalizability to a national cohort, heterogeneity in coding across MA contracts, even among switchers, warrants further scrutiny. In addition, as with any observational study, unobserved confounders that drive beneficiary choice of contract and reported diagnosis rates (eg, favorable selection) can bias comparisons between contracts. While the switchers design and controlling for proxies of pre-switch health mitigates confounding and reduces concerns about selection into MA on observed health, this study still depends on the assumption that, given market-level baseline health observed in TM, beneficiaries entering different MA contracts do not systematically differ in unobserved or future health characteristics in a manner correlated with subsequent coding. For example, if switchers choose contracts due to anticipated diagnoses not yet reflected in TM claims, the proposed approach would be unable to disentangle higher coding intensity in the anticipated diagnosis from selection into that particular contract. The proposed ranking does not measure total misreporting volume or aggregate overpayments, accounting for contract enrollment size only through shrinkage.

Ultimately, this analysis should be interpreted as a descriptive consistency check rather than gold-standard external validation, and emphasize that it does not distinguish diagnostic validity from contract-induced differences in treatment. Rankings should be interpreted as a prioritization based on relative coding intensity rather than definitive evidence of improper reporting.

To mitigate these limitations, the validation strategy aims to quantify similarity between the proposed ranking and prior approaches that examine relevant treatments as a proxy for diagnostic validity. While the impact of opting into Part D is mitigated by restriction to beneficiaries with a full year of Part D coverage, this design does not allow different contracts to affect Part D utilization differently. In addition, a simulation study is performed to evaluate robustness to ranking errors. Sample size limitations due to restriction to switchers and within-market estimation are mitigated through shrinkage toward the market-level mean. The matchup importance weights further dilute the influence of small markets or differences between contracts. The analysis focuses on claims submitted in 2019 since it preceded the COVID-19 pandemic and more recent post-COVID data was not available at the time of the study. While the resulting rankings may not be indicative of current rankings, the approach is general and can be applied to other/multiple years.

## Results

After applying exclusion criteria, the resultant cohort contains 128 455 Medicare beneficiaries who switched from TM into MA, enrolled in 427 MA contracts representing 2388 counties in 1894 markets. The median contract size in the cohort was 49 (IQR: 11.5-138.0) beneficiaries, each serving a median of 10 (IQR: 4.0-23.0) counties. The median age of beneficiaries was 72 years in 2018, with an interquartile range of 68 to 77 years (9 years). 55.2% of switchers were female, 84.4% were White, and switchers had a median HCC count of 1 (IQR: 0 to 2) and a pre-switch V22 CMS-HCC risk score of 0.64 (IQR: 0.35-1.08). See Table 1 for cohort details.

**Table 1:** Summaries of switchers’ cohort (*n* = 128 455 beneficiaries).

	*n* = 128 455
Median age (years, in 2018) (IQR)	72 (68—77)
% Female	55.2%
**Race/ethnicity**	
% White	84.4%
% Black	9.0%
% Hispanic	1.1%
% Asian	1.5%
% North American Native	0.3%
% Unknown/Other	3.9%
Avg. 2018 CMS-HCC risk score (IQR)	0.64 (0.35—1.08)
Median HCCs per beneficiary (IQR)	1 (0—2)

Source: Authors’ analysis of 2018 TM to 2019 MA switchers subject to exclusion criteria, 20% national sample of Medicare beneficiaries.

Abbreviations: CMS-HCC, Center for Medicare & Medicaid Services—Hierarchical Condition Categories (risk adjustment model name); HCC, Hierarchical Condition Categories; IQR, interquartile range; IQR, interquartile range.

Switcher-stayer differences are statistically significant due to the large sample size, but small in absolute terms (Appendix Table A2). Overall, compared to stayers, switchers were one year younger, 1.2% less likely to be female, and had a risk score of 0.11 points lower than stayers. The racial distribution differed slightly, but by no more than 1.1% in any cell.

**Figure 1:**
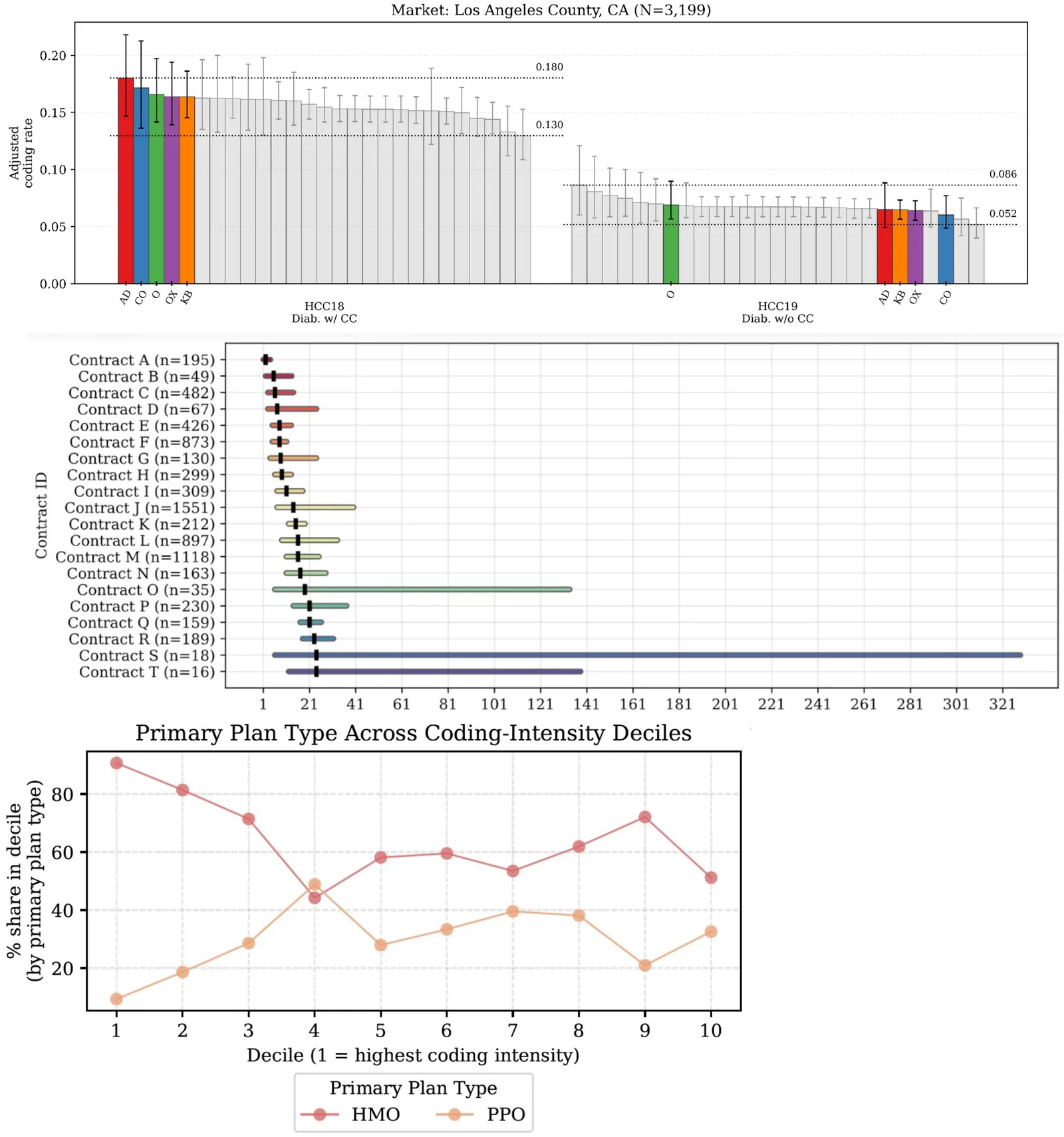
(a) (top) Local, shrinkage-based, and adjusted diagnosis rates for contracts (names anonymized) in Los Angeles County, CA for diabetes with (left) and without complications (right). Even within a market, heterogeneity in coding across contracts persists: estimated diagnosis rates range from 13.0% to 18.0% for HCC18 (diabetes *with* complications), while those for HCC19 (diabetes *without* complications) range from 5.2% to 8.6%. Furthermore, contracts that code most aggressively for HCC18 differ from those that code most aggressively for HCC19: the ordering of contracts by coding intensity shifts between HCC18 and HCC19. We aggregate such patterns across HCCs and across counties to produce the national ranking. Contract identifiers are anonymized using polyalphabetic labels (AD, CO, O, OX, KB). (b) (middle) Median national rank and IQR (width of the bars) across bootstrapped estimates (*n* = 1000) of relative coding intensity for top 20-ranked contracts (sorted by median national rank in ascending order), with number of switchers in each contract. A higher rank (further left) indicates more intense coding. Within-market rankings are aggregated into national per-contract rankings by fitting a weighted Bradley–Terry model. (c) (bottom) Share of contracts by primary plan type (HMO vs PPO) by decile (1 = highest coding intensity). The share of HMOs relative to PPOs decreases along the coding intensity ranking. Source: Results of authors’ proposed approach on switchers’ cohort (2018 TM to 2019 MA) and Medicare Advantage Plan Directory (Dec. 2019; part **c)** only.

In Los Angeles County, California (*N* = 3199), we observe substantial variation in market-level diagnosis rates for HCC19 (diabetes with complications) compared to HCC18 (diabetes without complications; Figure 1a). Note that Los Angeles County is the largest county in the switchers’ cohort by enrollment. Fitting a weighted Bradley-Terry model over per-market, per-HCC estimates across thousands of US counties and multiple HCCs subsequently yields a national per-contract ranking over 427 contracts, of which we present the top 20-ranked contracts with respect to estimated median national rank in Figure 1b. Bootstrapped error bars (*n* = 1000) represent sampling variability over independent runs of the ranking estimation procedure. The ranking over all 427 contracts is available in the supplement (Appendix Figures A5-A9). Because V28 of the HCC model is anticipated to reduce risk adjustment coding intensity, a ranking is also generated using V28 HCCs of the top 20 contracts in the supplement (Appendix Figure A10), finding that the V28 ranking exhibits wider bootstrap rank uncertainty than the corresponding V22 (Appendix Table A11).

As a summary of the rankings, we additionally plot the share of contracts by primary plan type (HMO or PPO) in Figure 1c for each decile of our ranking, defined based on whether a contract predominantly (>50%) offers HMOs or PPOs. The share of HMOs is largest in high-coding intensity deciles and decreases in favor of PPOs as coding intensity decreases.

### Internal validation

The Kendall tau correlation between our ranking and a national unadjusted ranking (observed per-contract mean CMS-HCC score) is 0.191 (empirical 95% CI: 0.158—0.223). The correlation increases to 0.249 (95% CI: 0.215—0.281) when comparing with a naive adjusted national ranking, where adjustment is performed identically to our within-market ranking, treating the national cohort as a single “market.” In the permutation test, error bars in the permuted cohort also widened compared to the original ranking, as expected: in the original ranking, the middle 50% of rank IQRs span 41 to 112 spots (9.6-26.2% of all contracts), while in the permuted ranking, the middle 50% of rank IQRs span 157 to 289 spots (36.8%-67.7% of all contracts). The permutation test's confidence intervals are also wider than those produced under the V28 ranking. A correlation matrix comparing these rankings is in Appendix Table A12.

### Partial external validation

**Figure 2:**
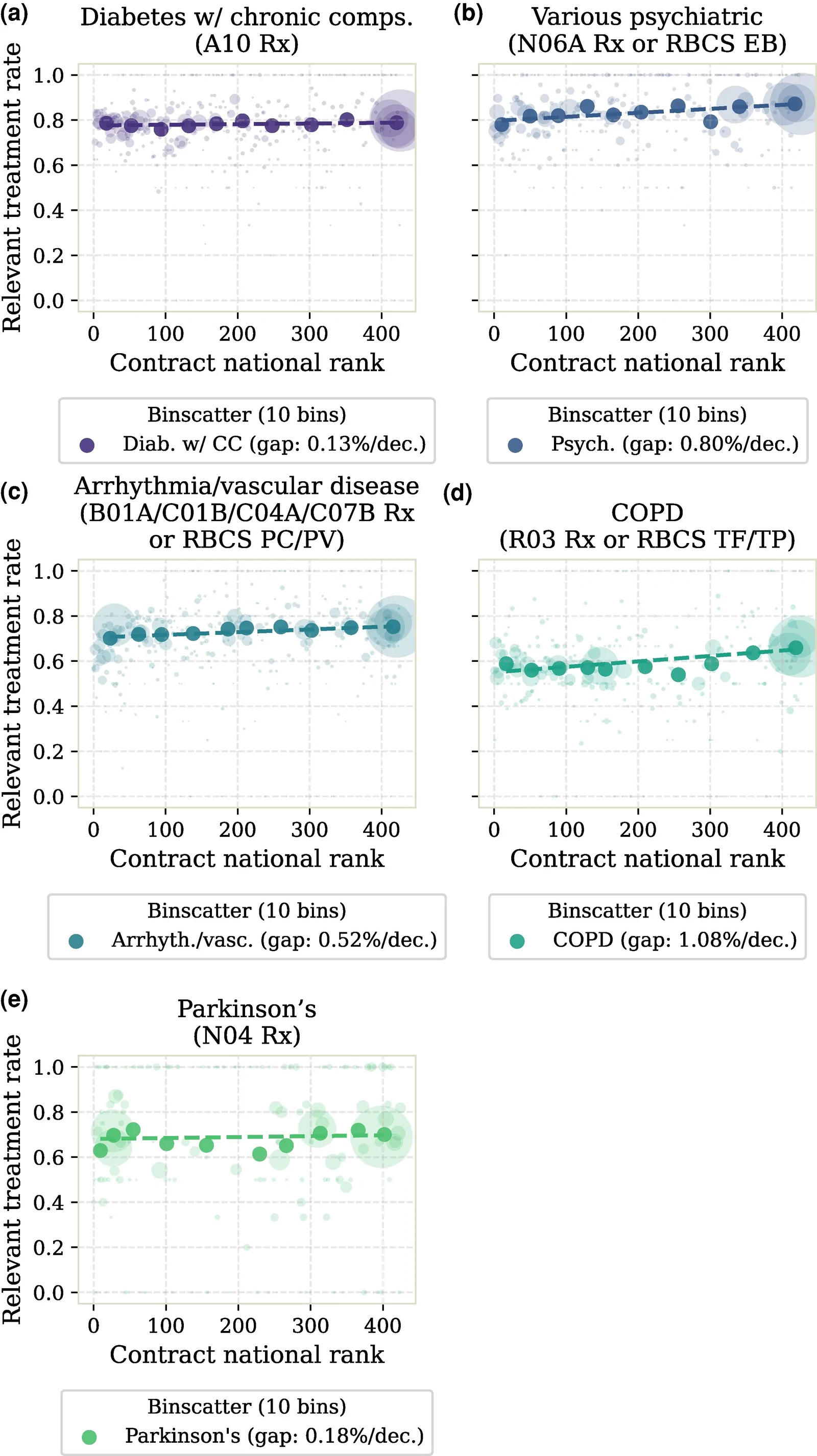
Binscatter plot of relevant treatments with respect to rankings computed using our approach over (a) diabetes with complications (HCC18), (b) various psychiatric conditions (HCC58), (c) arrhythmias/vascular disease (HCC96, 107, and 108), (d) COPD (HCC111), and (e) Parkinson's disease (HCC78). Transparent dots = treatment rate for a contract, where the size of the dot corresponds to the number of beneficiaries with the HCC in the contract. Solid dots = per-decile treatment rates, with an enrollment-weighted trendline (dashed) summarizing per-decile treatment rates. Source: Results of authors’ proposed approach on switchers’ cohort (2018 TM to 2019 MA), Medicare Advantage Part D Events and line files (2019), 2025 CMS Restructured BETOS Classification System file, NDC to RxCUI and RxCUI to ATC code mappings (Tuva Project).

For all 5 conditions analyzed, we observe positive gaps in relevant treatment rates across deciles of contracts computed with respect to ordinal rank in our national ranking (ie, decile 1 = higher relative coding intensity; Figure 2). These gaps range from 0.13% per decile (diabetes with complications) to 1.08% (COPD). Raw treatment rates and enrollment for each decile are reported in Appendix Tables A13-A17.

### Simulation study

**Figure 3:**
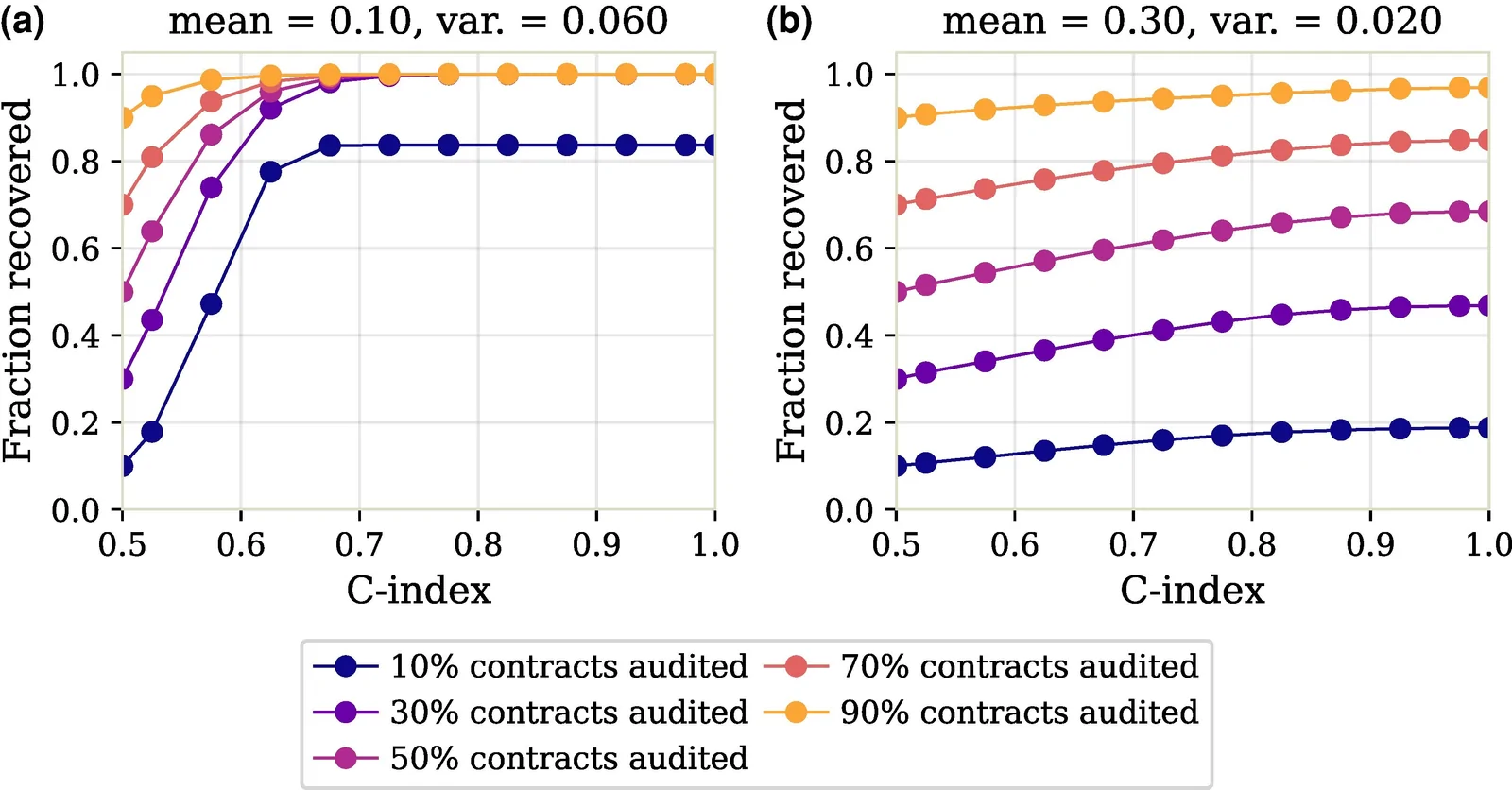
Graphs representing the fraction of misreported diagnoses audited as a function of the c-index (accuracy of a ranking) for different auditing intensities (10%, 30%, 50%, 70%, and 90%) and contract-level diagnosis rates. (a) displays the fraction of recovered diagnoses when the mean diagnosis rate is 0.1, and the spread of the diagnosis rate is 0.06; (b) displays the fraction of recovered diagnoses when the mean diagnosis rate is 0.3, and the spread of the diagnosis rate is 0.02. Source: Authors’ simulation study.

Our simulation indicates even an inaccurate ranking can be highly effective. Under a misreporting rate of 10% and variance of 6%, a moderately inaccurate rating (c-index: 0.65—0.70) can recover 83.6% of misreporting at a 10% audit rate (Figure 3a), while a random audit would only be expected to recover 10%. Further improvements to ranking accuracy yield marginal gains at a 10% audit intensity, as most of the top decile of worst offenders can already be identified. These gains diminish when misreporting rates are very high (30%) and low variance (2%): a similarly inaccurate ranking recovers fewer than 20% of misreporting, albeit a doubling over a random audit (Figure 3b). Among switchers, diagnosis rates are below <10% for all but the 5 most common HCCs (Appendix Table A2), suggesting that Figure 3a may be more realistic.

## Discussion

The proposed ranking approach for targeting MA program oversight has the potential to flag instances of high coding intensity and recover billions in overpayment for CMS. The approach is data-driven and does not require any assumptions on specific mechanisms of insurer-driven coding intensity beyond observed diagnosis rates. In simulation, even imperfect rankings substantially increase recovery rates when diagnosis prevalence is low, which applies to many HCCs. If high coding is concentrated in a few contracts, directing audits toward the highest-ranked contracts can recover a large share of potential excess payments with relatively few audits.

Several features of the proposed approach make it well-suited to this task. First, in contrast to prior work, this method does not make assumptions about specific mechanisms of coding intensity. Second, contracts are compared within markets, which corresponds with MA plan availability and naturally adjusts for geographic differences in clinical practice patterns and coding norms. Third, prior-year risk is incorporated to adjust for observed differences in beneficiary health status measured under TM, although unmeasured differences in health may remain. Finally, the method does not rely on anchoring MA coding rates to TM benchmarks or proxies for beneficiary health in TM. In particular, prior work has shown that payments in MA exceed those in TM even after adjustment for combinations of enrollee case-mix or geographical factors.^3,7,16^ Yet MAOs have historically responded to TM-anchored estimates with claims that potential errors in TM coding rates bias estimates of overspending.^18,19^ The proposed approach represents a data-driven middle ground: like other switcher-based approaches, the proposed approach trades population coverage for a common pre-enrollment coding baseline.^11,12^ Rather than estimating aggregate coding intensity, our choice to analyze contract-level heterogeneity in coding intensity allows direct usage of our approach as a prioritization tool for contract-level oversight.

Empirically, the resulting ranking aligns with established signals of differential coding intensity. The weak positive correlation between the proposed ranking and naïve rankings constructed with only national diagnosis rates suggests that the approach adds information beyond simple national measures. Rank stability deteriorates under permutation testing, reinforcing that the signal is not driven by noise. In addition, contracts dominated by HMOs tend to rank higher than PPO-dominated contracts, consistent with prior analyses documenting greater coding intensity in such plan structures.^9,16^ Higher-ranked contracts also exhibit lower rates of treatments relevant to reported diagnoses, echoing earlier findings.^10^ Additionally, the ranking generated from V28 rankings has confidence intervals that are wider than those from the V22 ranking, suggesting that V28 may partially mitigate coding incentives. However, the V28 ranking was computed on claims data that were collected when V22 HCCs largely determined risk adjustment, prior to the deployment of V28. V22 HCCs differ substantively from V28 HCCs, with only partial overlap between categories, and thus likely induce different coding incentives for MAOS. As a result, not all coding incentives associated with V28 are captured by the analyses (only those that also overlap with V22 HCCs). Notably, the permutation test still yielded wider rank confidence intervals than those produced using V28 HCCs, implying that some useful signal for prioritizing contracts for auditing may persist. We hypothesize that a similar analysis using data collected under V28 HCCs may yield a ranking with narrower confidence intervals, as MAOs may respond to new coding incentives.

A natural concern is that differences in coding intensity may reflect benign variation rather than inappropriate reporting. Wide uncertainty intervals across ranks also suggest that relative coding intensity of some contracts is inherently uncertain. Furthermore, since the ranking does not directly account for contract size, it is best used as a measure of relative coding intensity rather than total coding volume. This choice allows the approach to flag small contracts with high coding intensity, while preserving the option for CMS to incorporate enrollment-aware considerations. Given these tradeoffs, in practice, this ranking could be used to prioritize contracts above some enrollment threshold that rank highly with low uncertainty. Furthermore, while the usage of a TM-MA switchers’ cohort may constrain the generalizability of our ranking to the general MA population, risk scores for stayers may already be inflated due to insurers’ incentives to code more intensely.

### Policy implications

The relevant question for oversight is not whether every highly ranked contract is engaged in inappropriate coding, but whether directing audit resources toward such contracts improves audit yield relative to alternatives. In the presented ranking, higher-ranked contracts exhibit lower rates of treatments relevant to reported diagnoses, matching previous work.^10^ In simulation, even imperfect rankings substantially improve overpayment recovery compared to random audits when diagnosis rates are low (<10%), which holds for many HCCs. While changes to Medicare payment policy, such as redefinitions of reimbursement-eligible diagnoses, blanket payment reductions, or incorporation of patient health survey data, could temporarily mitigate coding intensity, insurers may identify new avenues for maximizing coding intensity.^11,20^ Approaches to audit prioritization that do not assume specific insurer strategies such as this ranking may remain robust even if MAOs update their coding strategies in response to policy changes.

## Conclusion

As CMS continues to expand auditing efforts in MA, methods to prioritize contracts with high coding intensity become increasingly critical. This work presents a data-driven approach to ranking MA contracts by coding intensity with the potential to substantially accelerate overpayment recovery, even allowing for some error in the rankings. The method independently recovers established signals of coding intensity, namely, gaps in treatment rates for conditions, without anchoring to TM spending or knowledge of potential coding intensity mechanisms. The results can inform audit prioritization strategies for CMS that streamline existing oversight processes and remain robust to shifts in MAO coding behavior.

## Supplementary Material

qxag209_Supplementary_Data
