# Supplementary material for: Streamlining oversight in Medicare advantage: measuring coding intensity via contract rankings
Source: Health Aff Sch. 2026 Sep 11;4(9):qxag209. doi: 10.1093/haschl/qxag209 (PMC13564226; doi:10.1093/haschl/qxag209)
Supplement: Supplementary file 1 [file qxag209_supplementary_data.zip › Rethinking_Coding_Intensity_Appendix.docx]

Appendix

Methods - Exclusion Criteria/Filtering

For the operationalization of the definitions used to define the switchers’ cohort, please see code located at: <https://github.com/MLD3/coding-intensity/>

Methods - Ranking Computation

We provide formal details on the ranking approach here: diagnosis rate estimation, adjustment, and rank aggregation.

To estimate diagnosis rates, we leverage the beta-binomial model to shrink estimates towards a per-market, per-stratum mean. Doing so shrinks extreme observed diagnosis rates stemming from low enrollment in specific strata. Without loss of generality, consider a single arbitrary diagnosis. We assume that diagnosis counts and rates $y_{c,m,s}$ and $p_{c,m,s}$, respectively, for a contract $c$ in market $m$ and stratum $s$of size $n_{c,m,s}$, are generated as follows:

$$y_{c,m,s} \sim Binom\left( n_{c,m,s}, p_{c,m,s} \right)$$

$$p_{c,m,s} \sim Beta\left( \alpha_{m,s}, \beta_{m,s} \right)$$

We estimate the per-stratum, per-market Beta parameters $\alpha_{m,s}$ and $\beta_{m,s}$using an empirical Bayes approach. Formally, we optimize

$$\underset{\alpha_{s},\beta_{s}}{\mathrm{argmax}} \sum_{j=1}^{J} \log P\left( y_{s,j}, n_{s,j} | \alpha_{s},\beta_{s} \right)$$

$$such that \frac{\alpha_{s}}{\alpha_{s}+ \beta_{s}}=\frac{\sum_{j=1}^{J} y_{s,j}}{\sum_{j=1}^{J} n_{s,j}} and 2\leq\alpha_{s}+\beta_{s}\leq\frac{\alpha_{s}\beta_{s}}{\varepsilon\left( \alpha_{s}+ \beta_{s} \right)^{2}}-1;\forall s.$$

i.e., we optimize the marginal log-likelihood of our data subject to the constraint that our prior mean matches the pooled per-market, per-stratum diagnosis rate, the constraint that $\alpha_{s}+\beta_{s}$ be at least 2 to avoid an U-shaped prior, and the constraint that $\alpha_{s}+\beta_{s}$ is bounded above as a variance lower bound to ensure identifiability if only one contract is present in a stratum. In practice, it is easier to leverage the reparameterization $\mu_{s}={\alpha_{s}}/\left( \alpha_{s}+\beta_{s} \right),\phi_{s}= \alpha_{s}+ \beta_{s}$ and optimize

$$\underset{\phi_{s}}{\mathrm{argmax}} \sum_{j=1}^{J} \log B\left( y_{s,j}+\mu_{s}\phi_{s}, n_{s,j}-y_{s,j}+\left( 1- \mu_{s} \right)\phi_{s} \right)-\log B\left( \mu_{s}\phi_{s}, \left( 1- \mu_{s} \right)\phi_{s} \right)$$

$$such that 2\leq\phi_{s}\leq\frac{\mu_{s}\left( 1- \mu_{s} \right)}{\varepsilon}-1;\forall s.$$

where $B\left( \cdot\right)$ is the Beta function. We take posterior mean of $y_{c,m,s}$ as our final diagnosis rate estimate, which is

$$E\left[ p_{c,m,s} \mid\alpha_{m,s}, \beta_{m,s} \right]= \frac{y_{c,m,s}+\alpha_{m,s}}{n_{c,m,s}+ \alpha_{m,s}+\beta_{m,s}}$$

To combine our per-stratum estimates into an adjusted per-market diagnosis rate $\hat{p}_{c,m}$, we leverage post-stratification and target a nationally representative population with per-stratum weights $w_{s}$:

$$\hat{p}_{c,m}=\sum_{s=1}^{S} w_{s}p_{c,m,s}$$

where $p_{c,m,s}$ is the posterior mean obtained in the last step, and $w_{s}$ is the proportion of beneficiaries in the national switchers’ cohort in the decile of interest.

After adjustment, we are left with independent, per-market “lists” of contract-level diagnosis rates. To aggregate these lists into a national ranking, we optimize a weighted version of the Bradley-Terry maximum likelihood objective using comparisons of adjusted diagnosis rates from MA contracts in the same market as input. We use boldface $\boldsymbol{\theta}$ to represent the latent “strength” parameter for each contract. The standard Bradley-Terry objective is

$$\underset{\boldsymbol{\theta}}{\mathrm{argmax}} \sum_{i<j} N_{ij}\log\frac{\theta_{i}}{\theta_{i}+\theta_{j}}+N_{ji}\log\frac{\theta_{j}}{\theta_{i}+\theta_{j}}$$

where $i$ and $j$ are contract indices, and $N_{ij}$ is the number of weighted “matchups” in which Contract $i$ has a higher adjusted diagnosis rate than Contract $j$, and $N_{ji}$is defined similarly:

$$N_{ij}\triangleq\sum_{m=1}^{M} \sum_{d=1}^{|HCC|} \omega_{m,i,j}^{\left( d \right)}\cdot\boldsymbol{1}\left[ \hat{p}_{i,m}^{\left( d \right)}>\hat{p}_{j,m}^{\left( d \right)} \right]$$

where $\omega_{m,i,j}^{\left( d \right)}$ is a matchup weight that depends on both contracts, the market, and the diagnosis. To mitigate the impact of small contracts in very few markets on the ranking, we add a term $\alpha\cdot\mathbb{E}\left[ \omega_{m,i,j}^{\left( d \right)} \right]$$ to the objective, equivalent to adding $\alpha$ pseudo-wins and pseudo-losses of “average” matchup weight to each comparison. Doing so regularizes win rates towards 50% with strength inversely proportional to the weight of “wins” in the observed data. This yields our final objective:

$$\underset{\boldsymbol{\theta}}{\mathrm{argmax}} \sum_{i<j} N_{ij}\log\frac{\theta_{i}}{\theta_{i}+\theta_{j}}+N_{ji}\log\frac{\theta_{j}}{\theta_{i}+\theta_{j}}+\alpha\mathbb{E}\left[ \omega_{m,i,j}^{\left( d \right)} \right]$$

We take $\alpha=5$ in our experiments. We define the weights to be

$$\omega_{m,i,j}^{\left( d \right)}=\left( \hat{p}_{i,m}^{\left( d \right)}-\hat{p}_{j,m}^{\left( d \right)} \right)\cdot HCC^{\left( d \right)}\cdot\frac{n_{m}}{\sum_{m=1}^{M} n_{m}}$$

where $HCC^{\left( d \right)}$ is the CMS-HCC V22 model weight for condition category $d$ in the non-dual aged cohort, and $n_{m}$is the number of beneficiaries in our cohort in market $m$. Intuitively, we weight matchups by the “margin of victory,” the CMS-HCC weight, and the overall market size. Doing so means that “wins” by a large margin, in high-leverage HCCs, in larger markets, have a greater impact on the ranking than smaller wins, wins in low-reimbursement HCCs, or small markets. The optimization proceeds by using rank centrality with a PageRank-style damping factor as an initialization to Hunter’s MM, a standard approach for optimizing the Bradley-Terry model.^1–3^

Methods – Computing Treatment Rates

For the treatment gap analysis, the treatment rate is computed as the proportion of beneficiaries who received a relevant procedure or filled a relevant prescription at any time during 2019 (i.e., their first year on MA). Thus, we define treatment receipt at the beneficiary level using claims data. A beneficiary is classified as having received a given treatment if either condition holds:

- any reimbursement-eligible claim contains a Healthcare Common Procedure Coding System (HCPCS) code that maps to the relevant Restructured BETOS Classification System (RBCS) category, or
- beneficiary filled a prescription with a National Drug Code (NDC) that maps to a relevant Anatomical Therapeutic Chemical (ATC) category.

Mappings from HCPCS to RBCS are obtained via the 2025 CMS RBCS release.^4^ Mappings from NDC to ATC codes are obtained via an intermediate mapping to RxCUI codes; both mappings for prescription drugs (NDC to RxCUI, RxCUI to ATC) are sourced from the open-source Tuva Project. See Appendix Figure A2 for all such RBCS and ATC codes.^5^

Methods - Simulation Study

To investigate the efficacy of our ranking method, we conduct a simulation study. At a high level, we simulate the process of auditing contracts following different rankings. We generate rankings with varying levels of accuracy as measured by the c-index across a variety of distributions describing simulated per-contract adjusted diagnosis misreporting rates. The c-index is a measure of

Formally, we draw per-contract diagnosis rates from a Beta distribution. We use a mean-variance parameterization of the Beta distribution $\mathrm{Beta}\left( \mu,\sigma^{2} \right)$ where $\mu=\frac{\alpha}{\alpha+\beta}$ and $\sigma^{2}=\frac{\alpha\beta}{\left( \alpha+\beta\right)^{2}(\alpha+\beta+1)}$ in terms of the standard $\alpha,\beta$-parameterization. We sweep through $\mu\in\left\{ 0.1, 0.2, 0.3, 0.4, 0.5, 0.6, 0.7, 0.8, 0.9 \right\}$ and

$\sigma^{2}\in\left\{ 0.020, 0.025, 0.030, 0.035, 0.040, 0.045, 0.050, 0.055 \right\}$ and draw 427 per-contract diagnosis misreporting rates, matching the number of contracts in the switchers’ cohort. These values stand in for the true adjusted misreporting rate for an arbitrary HCC for each contract.

To evaluate the efficiency of our ranking, we compute *audit recovery curves* on simulated noisy rankings with varying levels of accuracy. The audit recovery curve is defined as the cumulative proportion of misreported diagnoses audited as a function of the number of contracts audited when following a ranking of contracts. As a ranking accuracy metric, we use the c-index, which corresponds to the percentage of contract pairs ranked in the same direction between the two rankings. In our setting, where all contracts are pairwise comparable, the c-index is equivalent to Kendall’s tau + 1 divided by two between the predicted and true ranking.

We simulate 1000 rankings with c-index in [0.50, 0.55], [0.55, 0.60], …, [0.95, 1] by adding mean-zero isotropic Gaussian noise to the sampled diagnosis misreporting rates from the Beta distribution described above. Note that we do not clip the outputs since we are merely interested in obtaining a noisy ranking. We adaptively set the noise as follows to ensure that ranking accuracy values span the entirety of the c-index bin.

More formally, for each c-index bin with bounds $\left[ c_{l}, c_{h} \right]$, we begin with an input vector of diagnosis misreporting rates $\boldsymbol{v}$ and variance $\sigma_{t}$ for some timestep t. The vector $\boldsymbol{v}$ stands in for a “perfect” ranking. Then, we generate a noisy ranking $\tilde{\boldsymbol{v}}=\boldsymbol{v}+\varepsilon$, where $\varepsilon\sim N(0, \sigma_{t}^{2}\cdot\boldsymbol{I}$). We compute the c-index of $\tilde{\boldsymbol{v}}$ with respect to $\boldsymbol{v}$, which we call $C\left( \boldsymbol{v,}\tilde{\boldsymbol{v}} \right)\boldsymbol{.}$If $C\left( \boldsymbol{v,}\tilde{\boldsymbol{v}} \right)\boldsymbol{<}c_{l}$, our noisy ranking has a c-index that lies below the target bin, so we *reject* the sample $\tilde{\boldsymbol{v}}$ and decrease the noise level: $\sigma_{t+1}:=\sigma_{t}/(1+\alpha_{t})$, where $\alpha_{t}$is an adaptive step size parameter. Likewise, if $C\left( \boldsymbol{v,}\tilde{\boldsymbol{v}} \right)\boldsymbol{>}c_{h}$, our noisy ranking has a c-index above the target bin, so we similarly *reject*$\tilde{\boldsymbol{v}}$ and increase the noise level $\sigma_{t+1}:=\sigma_{t}*(1+\alpha_{t})$.

If $c_{l}<C\left( \boldsymbol{v,}\tilde{\boldsymbol{v}} \right)\boldsymbol{<}c_{h}$, our noisy ranking has a c-index that lies within the target bin. Thus, we *accept* $\tilde{\boldsymbol{v}}$ and adjust $\alpha_{t}=\alpha_{0}e^{-5 \bar{C}}$ where  $\bar{C}=\frac{c_{l}+c_{h}}{2}$is the midpoint of the c-index bin being targeted. Then, we compute the sample quartiles $Q_{1}$and$Q_{3}$ and compute absolute distances $\Delta_{l}$ and $\Delta_{h}$ to $c_{l}$ and $c_{h}$, respectively. If $\Delta_{l}>1.5\Delta_{h}$, then the c-indices in the current bin skew high and we update $\sigma_{t+1}:=\sigma_{t}*(1+\alpha_{t})$. Otherwise, if $\Delta_{h}>1.5\Delta_{l}$, the c-indices in the current bin skew low and we update $\sigma_{t+1}:=\sigma_{t}/(1+\alpha_{t})$. If neither holds, $\sigma_{t}$ is left unchanged.

Supplementary References

1.	Page L, Brin S, Motwani R, Winograd T. *The PageRank Citation Ranking: Bringing Order to the Web.* Stanford InfoLab; 1999. http://ilpubs.stanford.edu:8090/422/

2.	Negahban S, Oh S, Shah D. Rank Centrality: Ranking from Pairwise Comparisons. *Oper Res*. 2017;65(1):266-287. doi:10.1287/opre.2016.1534

3.	Hunter DR. MM algorithms for generalized Bradley-Terry models. *Ann Stat*. 2004;32(1). doi:10.1214/aos/1079120141

4.	Centers for Medicare & Medicaid Services. Restructured BETOS Classification System (RBCS) dataset. Published online 2025. https://data.cms.gov/provider-summary-by-type-of-service/provider-service-classifications/restructured-betos-classification-system

5.	Tuva Health Team. The Tuva Project. Published online 2026. https://github.com/tuva-health/tuva

**Figure A1: Full cohort diagram of the switchers’ cohort (2018 TM to 2019 MA).**

**Table A2: Summary statistics of switchers’ cohort (n = 128,455 beneficiaries, left) and MA-to-MA stayers (n = 2,302,699, right) with respect to age, sex, racial distribution, average CMS-HCC risk score, and HCC count.**

|  | TM to MA Switchers  (n = 128,455) | MA stayers  (n = 2,302,699) | p-value |
| --- | --- | --- | --- |
| Median Age (years, in 2018) (IQR) | 72 (68 - 77) | 73 (69 - 79) | <.001 |
| % Female | 55.2% | 56.4% | <.001 |
| Race/Ethnicity |  |  | <.001 |
| % White | 84.4% | 83.3% |  |
| % Black | 9.0% | 8.8% |  |
| % Hispanic | 1.1% | 1.4% |  |
| % Asian | 1.5% | 2.2% |  |
| % NA Native | 0.3% | 0.2% |  |
| % Unknown/Other | 3.9% | 4.2% |  |
| Avg. 2018 CMS-HCC risk score (IQR) | 0.64 (0.35 - 1.08) | 0.75 (0.43 - 1.28) | <.001 |
| Median HCCs per beneficiary (IQR) | 1 (0 - 2) | 1 (0 - 3) | <.001 |

**Table A3: Eligible HCCs (prevalence > 0.5%) in switchers’ cohort, sorted in descending order of prevalence.**

| **HCC (Version 22)** | **Description** | **Count (% prevalence)** |
| --- | --- | --- |
| HCC18 | Diabetes with Chronic Complications | 22,622 (17.61%) |
| HCC108 | Vascular Disease | 20,977 (16.33%) |
| HCC96 | Specified Heart Arrhythmias | 18,143 (14.12%) |
| HCC111 | Chronic Obstructive Pulmonary Disease | 15,277 (11.89%) |
| HCC85 | Congestive Heart Failure | 14,965 (11.65%) |
| HCC58 | Major Depressive, Bipolar, and Paranoid Disorders | 12,235 (9.52%) |
| HCC22 | Morbid Obesity | 10,286 (8.01%) |
| HCC19 | Diabetes without Complication | 10,071 (7.84%) |
| HCC40 | Rheumatoid Arthritis and Inflammatory Connective Tissue Disease | 8,443 (6.57%) |
| HCC12 | Breast, Prostate, and Other Cancers and Tumors | 8,394 (6.53%) |
| HCC48 | Coagulation Defects and Other Specified Hematological Disorders | 7,121 (5.54%) |
| HCC23 | Other Significant Endocrine and Metabolic Disorders | 4,790 (3.73%) |
| HCC135 | Acute Renal Failure | 4,718 (3.67%) |
| HCC88 | Angina Pectoris | 4,355 (3.39%) |
| HCC84 | Cardio-Respiratory Failure and Shock | 3,458 (2.69%) |
| HCC55 | Drug/Alcohol Dependence | 2,931 (2.28%) |
| HCC100 | Ischemic or Unspecified Stroke | 2,911 (2.27%) |
| HCC11 | Colorectal, Bladder, and Other Cancers | 2,335 (1.82%) |
| HCC2 | Septicemia, Sepsis, Systemic Inflammatory Response Syndrome/Shock | 2,292 (1.78%) |
| HCC161 | Chronic Ulcer of Skin, Except Pressure | 2,200 (1.71%) |
| HCC79 | Seizure Disorders and Convulsions | 2,138 (1.66%) |
| HCC107 | Vascular Disease with Complications | 2,137 (1.66%) |
| HCC124 | Exudative Macular Degeneration | 2,055 (1.60%) |
| HCC86 | Acute Myocardial Infarction | 1,921 (1.50%) |
| HCC10 | Lymphoma and Other Cancers | 1,836 (1.43%) |
| HCC47 | Disorders of Immunity | 1,727 (1.34%) |
| HCC75 | Myasthenia Gravis/Myoneural Disorders and Guillain-Barre Syndrome/Inflammatory and Toxic Neuropathy | 1,709 (1.33%) |
| HCC78 | Parkinson's and Huntington's Diseases | 1,678 (1.31%) |
| HCC176 | Complications of Specified Implanted Device or Graft | 1,552 (1.21%) |
| HCC21 | Protein-Calorie Malnutrition | 1,538 (1.20%) |
| HCC103 | Hemiplegia/Hemiparesis | 1,411 (1.10%) |
| HCC35 | Inflammatory Bowel Disease | 1,402 (1.09%) |
| HCC8 | Metastatic Cancer and Acute Leukemia | 1,360 (1.06%) |
| HCC33 | Intestinal Obstruction/Perforation | 1,343 (1.05%) |
| HCC112 | Fibrosis of Lung and Other Chronic Lung Disorders | 1,309 (1.02%) |
| HCC87 | Unstable Angina and Other Acute Ischemic Heart Disease | 1,305 (1.02%) |
| HCC115 | Pneumococcal Pneumonia, Empyema, Lung Abscess | 1,222 (0.95%) |
| HCC9 | Lung and Other Severe Cancers | 1,197 (0.93%) |
| HCC169 | Vertebral Fractures without Spinal Cord Injury | 1,143 (0.89%) |
| HCC170 | Fracture/Dislocation | 1,018 (0.79%) |
| HCC137 | Chronic Kidney Disease, Severe (Stage 4) | 1,001 (0.78%) |
| HCC188 | Artificial Openings for Feeding or Elimination | 899 (0.70%) |
| HCC122 | Proliferative Diabetic Retinopathy and Vitreous Hemorrhage | 874 (0.68%) |
| HCC39 | Bone/Joint/Muscle Infection/Necrosis | 825 (0.64%) |
| HCC189 | Amputation Status, Lower Limb/Amputation Complications | 655 (0.51%) |

**Table A4: HCC to treatment (ATC Level II drug categories or RBCS procedure categories) mappings used for treatment gap analysis.**

| **HCC(s)** | **ATCs/procedures** |
| --- | --- |
| Diabetes with complications (HCC 18) | ATC A10 |
| Major Depressive, Bipolar, and Paranoid Disorders (HCC58) | ATC N06A; RBCS EB |
| Specified Heart Arrhythmias, Vascular Disease with Complications, Vascular Disease (HCC96, 107, 108) | ATC B01A, C01B, C07A, C04A; RBCS PC |
| Chronic Obstructive Pulmonary Disease (HCC111) | ATC R03; RBCS TF, TP |
| Parkinson's and Huntington's Diseases (HCC78) | ATC N04 |

**Figure A5: Contracts ranking 1st to 90th (inclusive) in switchers’ cohort, (2018 TM to 2019 MA).**

**Figure A6: Contracts ranking 91st to 180th (inclusive) in switchers’ cohort, (2018 TM to 2019 MA).**

**Figure A7: Contracts ranking 181st to 270th (inclusive) in switchers’ cohort, (2018 TM to 2019 MA).**

**Figure A8: Contracts ranking 271st to 360th (inclusive) in switchers’ cohort, (2018 TM to 2019 MA).**

**Figure A9: Contracts ranking 361st to 427th (inclusive) in switchers’ cohort, (2018 TM to 2019 MA).**

**Figure A10: Median rank and IQR across 1000 bootstrap samples of the switchers cohort of coding intensity rank for top 20-ranked contracts according to V28 HCCs.**

**Table A11:**  Comparison of bootstrap widths for (from left to right) the ranking using all V22 HCCs, the permuted ranking using all V22 HCCs, the ranking using all V28 HCCs, and the permuted ranking using all V28 HCCs.

|  | V22 | | V28 | |
| --- | --- | --- | --- | --- |
|  | All HCCs | Permuted | All HCCs | Permuted |
| median | 67.25 | 223.00 | 83.00 | 218.25 |
| IQR | 66.62 | 134.62 | 92.62 | 121.63 |

**Table A12: Correlation matrix (Kendall’s tau) between different rankings with empirical 95% CI across bootstraps.**

|  | **Ours** | **No market splitting (national monomarket)** | **No BT (adjusted CMS-HCC score)** | **No adjustment (observed CMS-HCC score)** |
| --- | --- | --- | --- | --- |
| **Ours** | 1 | 0.196 (0.157, 0.239) | 0.249 (0.215, 0.281) | 0.191 (0.158, 0.223) |
| **No market-splitting (national monomarket)** |  | 1 | 0.409 (0.375, 0.443) | 0.276 (0.248, 0.303) |
| **No BT ( adjusted CMS-HCC score)** |  |  | 1 | 0.468 (0.442, 0.494) |
| **No adjustment ( observed CMS-HCC score)** |  |  |  | 1 |

**Table A13: Raw rates of relevant treatments by contract decile for diabetes with complications (HCC18). ATC A10: Drugs for diabetes. Deciles correspond to ordinal rank (1 = highest relative coding intensity; 10 = lowest relative coding intensity).**

| **Decile** | **Treatment rate (ATC A10 prescription)** | **Total enrollment** |
| --- | --- | --- |
| 1 | 78.5% | 15,496 |
| 2 | 77.5% | 13,336 |
| 3 | 75.9% | 7,048 |
| 4 | 77.5% | 8,286 |
| 5 | 78.4% | 3,373 |
| 6 | 79.7% | 4,757 |
| 7 | 77.5% | 2,348 |
| 8 | 77.9% | 3,389 |
| 9 | 80.2% | 2,018 |
| 10 | 78.8% | 64,049 |

**Table A14: Raw rates of relevant treatments by contract decile for major depressive, bipolar, and paranoid disorders (HCC58). ATC N06A: Antidepressants. RBCS EB: Evaluation & Management, Behavioral Health. Deciles correspond to ordinal rank (1 = highest relative coding intensity; 10 = lowest relative coding intensity).**

| **Decile** | **Treatment rate (ATC N06A prescription or RBCS EB code)** | **Total enrollment** |
| --- | --- | --- |
| 1 | 78.0% | 12,170 |
| 2 | 81.8% | 10,966 |
| 3 | 82.0% | 10,016 |
| 4 | 86.1% | 3,124 |
| 5 | 82.3% | 6,045 |
| 6 | 83.5% | 3,208 |
| 7 | 86.3% | 2,826 |
| 8 | 79.3% | 4,411 |
| 9 | 86.0% | 15,824 |
| 10 | 87.1% | 55,064 |

**Table A15: Raw rates of relevant treatments by contract decile for specified heart arrhythmias, vascular disease with complications, and vascular disease (HCC96, 107, and 108). ATC B01A: Antithrombotic agents, C01B: Antiarrhythmics, class I and III, C07A: Beta-blocking agents, C04A: Peripheral vasodilators; RBCS PC: Procedure - Cardiovascular. Deciles correspond to ordinal rank (1 = highest relative coding intensity; 10 = lowest relative coding intensity).**

| **Decile** | **Treatment rate (ATC B01A/C01B/C07A/C04A  prescription or RBCS PC code)** | **Total enrollment** |
| --- | --- | --- |
| 1 | 70.1% | 27,222 |
| 2 | 71.9% | 7,925 |
| 3 | 71.8% | 6,238 |
| 4 | 72.3% | 2,714 |
| 5 | 74.2% | 5,830 |
| 6 | 74.7% | 4,621 |
| 7 | 75.2% | 2,411 |
| 8 | 73.6% | 7,734 |
| 9 | 74.8% | 5,682 |
| 10 | 75.3% | 53,795 |

**Table A16: Raw rates of relevant treatments by contract decile for chronic obstructive pulmonary disease (HCC111). ATC R03: Drugs for obstructive airway diseases. RBCS TF/TP: pulmonary function tests. Deciles correspond to ordinal rank (1 = highest relative coding intensity; 10 = lowest relative coding intensity).**

| **Decile** | **Treatment rate (ATC R03 prescription or RBCS TF/TP code)** | **Total enrollment** |
| --- | --- | --- |
| 1 | 58.9% | 14,987 |
| 2 | 56.0% | 9,463 |
| 3 | 56.8% | 8,974 |
| 4 | 57.1% | 6,424 |
| 5 | 56.4% | 15,090 |
| 6 | 57.5% | 2,666 |
| 7 | 54.0% | 2,158 |
| 8 | 58.8% | 6,053 |
| 9 | 63.7% | 2,569 |
| 10 | 65.9% | 55,354 |

**Table A17: Raw rates of relevant treatments by contract decile for Parkinson’s and Huntington’s diseases. ATC N04: Anti-parkinson drugs.** **Deciles correspond to ordinal rank (1 = highest relative coding intensity; 10 = lowest relative coding intensity).**

| **Decile** | **Treatment rate (ATC N04 prescription)** | **Total enrollment** |
| --- | --- | --- |
| 1 | 62.9% | 3,308 |
| 2 | 69.7% | 29,460 |
| 3 | 72.2% | 1,952 |
| 4 | 66.0% | 4,211 |
| 5 | 65.2% | 1,989 |
| 6 | 61.4% | 4,582 |
| 7 | 65.1% | 7,822 |
| 8 | 70.6% | 18,030 |
| 9 | 71.9% | 6,264 |
| 10 | 70.0% | 38,561 |
